# Extracorporeal membrane oxygenation to support repeated whole-lung lavage in a patient with pulmonary alveolar proteinosis in life threatening dyspnoe – a case report

**DOI:** 10.1186/s12871-015-0152-3

**Published:** 2015-11-30

**Authors:** Martina Krecmerova, Frantisek Mosna, Vladimír Bicek, Frantisek Petrik, Alzbeta Grandcourtova, Marek Lekes, Tomas Vymazal

**Affiliations:** 1 Department of Anaesthesiology and ICM, 2nd Faculty of Medicine, Charles University in Prague, Prague, V Úvalu 84, 150 06 Praha 5 Czech Republic; 2 Department of Anaesthesiology and ICM, 2nd Faculty of Medicine, Motol University Hospital, Prague, V Úvalu 84, 150 06 Praha 5 Czech Republic; 3 Pneumology Department, 2nd Faculty of Medicine, Charles University in Prague, Prague, V Úvalu 84, 150 06 Praha 5 Czech Republic; 4 Department of Rehabilitation and Sports Medicine, 2nd Faculty of Medicine, Charles University in Prague, Prague, V Úvalu 84, 150 06 Praha 5 Czech Republic

**Keywords:** Pulmonary alveolar proteinosis (PAP), Whole-lung lavage (WLL), Extracorporeal membrane oxygenation (ECMO), General anesthesia (GA)

## Abstract

**Backgroud:**

Pulmonary alveolar proteinosis is a rare disorder that is characterized by a large accumulation of lipoproteinaceous material within the alveoli. This causes respiratory failure due to a restriction of gas exchange and changes in the ventilation/perfusion ratio. Clinical symptoms are variable and depend on the severity of damage of the lung parenchyma. Treatment method is whole-lung lavage, where the accumulated lipoproteinaceous material is removed using large quantities of saline.

**Case presentation:**

This case report describes a 45 year old patient with advanced pulmonary alveolar proteinosis. Due to the presence of severe global respiratory insufficiency, this patient could not undergo the classic whole-lung lavage using a double-lumen tube and selective lung ventilation. The whole-lung lavage was performed with the support of veno-venous extracorporeal membrane oxygenation. A total of 27 l of warm saline was used.

**Conclusion:**

According to the current published literature, whole-lung lavage with extra-corporeal membrane oxygenation support is a very rare treatment method. Even when taking into account all of the risks associated with whole-lung lavage and v-v extracorporeal membrane oxygenation support, we found that this technique is very effective and, without a doubt, it saved the life of our patient.

## Background

Pulmonary alveolar proteinosis (PAP) is a rare disorder first described by Rosen and colleagues in 1958 [1]. This disease is characterized by an accumulation of phospholipoproteinaceous material inside the alveoli due to a disruption in surfactant homeostasis. The prevalence of PAP is estimated to be around 4 cases per 1 million. Males, smokers and persons between 30 and 50 years are most commonly affected. Symtoms include shortness of breath (dyspnea), cough and, in one third of cases, nail clubbing. Opportunistic bacterial and mycotic infections are common complications. There is a variable interindividual progression of this disease which can range from spontaneous recovery to terminal cardiorespiratory failure. Diagnosis is based on the results from bronchoalveolar lavage (BAL), histology and immunohistochemical tests, on the presence of granulocyte-macrophage colony stimulating factor (GM-CSF) antibodies and computer tomography (CT) or high resolution computer tomography (HRCT) results which typically shows areas of patchy ground-glass opacification and interlobular thickening, which together produce the “crazy paving pattern” [2, 3].

PAP can occur as an acquired disease (primary or idiopathic PAP) and then is characterized by the production of GM-CSF antibodies, therefore is autoimmune in origin. Congenital forms of PAP are less commonly seen and they are caused by a mutated gene responsible for surfactant production or by a mutated receptor for GM-CSF. These forms can be treated with medical therapy (GM-CSF substitutes, biological treatment with monoclonal antibodies or plasmapheresis). Secondary forms of PAP are associated with other illnesses, mainly hematooncological diseases, exposure to anorganic materials (eg. silicone), or from side-effect of medication such as immunosuppresive drugs or amiodarone. Rarely, PAP can be a part of the aquired immune deficiency syndrome (AIDS).

The treatment of aquired forms of PAP are mainly bronchoalveolar lavage where the lungs are flushed out with large quantities of saline. This can be performed on individual lung segments, lobes or on the whole lung with possibility for longer pauses between each lavage treatment. The whole-lung lavage (WLL) has been described as the most effective method of treatment [2, 3].

## Case presentation

This is a case report of a 45-year old female with a history of work exposure to a dusty environment. She was living on a farm where the hens, rabbits and dogs were present and also had a history of smoking 20 cigarettes a day for 19 years. The patient reported progressive worsening of the shortness of breath on exertion for the last year. A diagnosis of PAP was performed based on the HRCT and BAL results. One-sided lung lavage was repeatedly performed with the final procedure being complicated by acute respiratory failure and the need for mechanical ventilation. The therapeutic effect of the lavage treatments was short-lived and was followed by progression of the disease. Oxygen saturation on room air at rest (SpO2) was measured as low as 80 %. The patient was therefore dependent on a long-term home oxygen therapy. One year following the diagnose of PAP, SpO2 at rest on room air dropped to 72 %. The patient’s health status began to deteriorate, withan insufficiency of the right heart developing and a Mycobacterium intracellular lung infection was diagnosed and was subsequently repeatedly treated with a combination of antibodies. High resolution computed tomography results showed a severe ‘crazy paving’ pattern bilaterally. Arterial blood gas on home oxygen therapy with O2 at 6L/min showed pO2 6.36 kPa and pCO2 3.6 kPa. Due to rapid disease progression and the presence of severe respiratory insufficiency, the patient was indicated for the bilateral lung lavage and the procedure was performed using veno-venous ECMO support. The protocol recommended by the American College of Chest Physicians from 2009 was followed during the performance of this treatment [4].

Upon admission to the operating theatre, non-invasive monitoring was commenced (electrocardiograph - ECG, non-invasive blood pressure measurement – NIBP, pulse oximetry). The initial values were as follows – sinus rhythm 90/min, NIBP 160/90 mmHg, SpO2 ( on the room air ) was 80 %. An 18 G peripheral venous cannula was placed to the right cubital vein. Induction to general anesthesia was commenced with remifentanil at 0.25 ug/kg/min, 180 mg of propofol and 70 mg of rocuronium. Tracheal intubation was performed with a 37 Ch left-sided double-lumen tube without a carinal hook (Robertshaw) and its position was confirmed with a bronchoscope. A urinary catheter was inserted. The patient was ventilated using volume-controlled ventilation (VCV) with a tidal volume of 480 mL during bilateral lung ventilation, a respiratory rate of 10 breaths/min, an inspiration:expiration ratio of 1:2, positive-end expiratory pressure (PEEP) of 5 cm H2O and the initial FiO2 was 1.0. Ventilation was adjusted based on the SpO2 and ETCO2 values until the introduction of the v-v ECMO. Anesthesia was maintained intravenously (TIVA) with propofol at a dosage of 0.7 mcg/Kg/min and analgesia with remifentanil at a dosage of 0.16 mcg/kg/min. Based on the recommendation of the Local Hospital Drug Commitee, the procedure was performed with antibiotic prophylaxis using 2g of cefotaxim 6 hourly.

After examination of both internal jugular veins and femoral veins, a 8.5 Fr x 20 cm central venous catheter was insered into the left internal jugular vein. Additionally, a 8.5 Fr x 10 cm sheath was placed into the left subclavian vein to allow for potential invasive monitoring using a pulmonary catheter or in the instance that high-volume fluid therapy would be necessary. Arterial cannulation was performed on the left radial artery to ensure direct blood pressure monitoring.

Selective ventilation of the right or left lung was not possible due to the production of a right-left shunt which would lead to the severe derangement of oxygenation and highly increase the accumulation of CO2 in the body.

Using transesophaegeal echocardiography (TEE), the kinetics of the left and right heart were found to be normal without any valve pathology or pericardial separation. A bolus of heparin (5000 I.U.) was administered prior to cannulation for the v-v ECMO. The infusion cannula was placed in the right internal jugular vein while the drainage cannula was placed in the right femoral vein. The position of the both infusion cannulas was confirmed using TEE. The flow through ECMO was set at 4837 mL/min and was determined after calculating the patient’s body surface area which was 1.79m2. After reaching a flow of 4500 mL/min on the ECMO with a FiO2 of 0.8, then the parametres of mechanical ventilation were adjusted accordingly (tidal volume - Vt 300-350 ml, respiratory frequency - RF 10 per minute, PEEP 5 cmH2O, FiO2 0,4). Body core temperature was measured in the esophagus. The activated clotting time (ACT) was measured at precise intervals and heparin boluses were given every 160-200 s.

**Fig. 1:**
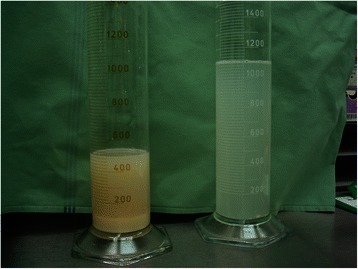
The character of the first (on the left) and the last portions of fluid used for the lung lavage

After fixation of the ECMO cannulae, the patient was positioned on her right side. The position of the DLT was checked with a bronchoscope. Prior to insertion, both cuffs were tested under water for any leakage. The operating table was placed in anti-Trendelenburg (“head up”) position. Subsequently, the first 1000 mL of warmed (37 °C) saline was introduced into the left lung, after which followed a 4 min long manual physiotherapeutic massage of the chest wall – using percussional postural massage and vibrational techniques with the goal of freeing the maximal amount of accumulated material. Afterwards, the fluid was released with the help of gravity by placing the patient in the Trendelenberg position (“head down”). This procedure was repeated several times. At first, the collected fluid was yellowish-white and cloudy with floccules that sedimented and were collected. Progressively, the fluid became more and more clear (Fig. 1).

After completing the lavage of the left lung, the patient was positioned on her left side and the same procedure was performed on the right lung. Over 225 min, the left lung was rinsed with 12,000 mL of warm saline and 11,680 mL of the fluid was collected (320 mL were absorbed) and over the course of 150 min, the right lung was rinsed with 15,000 mL of warm saline and 14,530 mL were collected (470 mL were absorbed). During the entire procedure, several incidents of isolated hemodynamically insignificant supraventricular and polytopic ventricular ectopics were registered but did not require any intervention. Normothermia was maintained for the duration of the procedure. Electrolyte levels were monitored throughout the procedure and the levels of potassium and magnesium had to be corrected. Diuresis was supported using furosemide at a dosage of 15 mg/h. In order to ensure proper perfusion, mean arterial pressure (MAP) was maintained at 75 mmHg by continuous administration of norepinephrine at a dosage of 0.10-0.15 ug/kg/min.

Oxygenation parameters during the procedure which lasted 9 h are summarized at the Table 1.

**Table 1:** Oxygenation parameters during the procedure

Initial value	FiO2 0.21	paO2 6.36 kPa	SaO2 85 %, SpO2 80 %
After induction	FiO2 1.0		SpO2 93 %
v-v ECMO start	FiO2 0.8	paO2 39.78 kPa	SaO2 100 %
After 90 min of left lung lavage, v-v ECMO	FiO2 0.6	paO2 16.16 kPa	SaO2 98 %
225 min – end time of left lung lavage, v-v ECMO	FiO2 0.8	paO2 11.59 kPa	SaO2 95,5 %
Next 150 min - end time of right lung lavage, v-v ECMO	FiO2 0.8	paO2 13,62 kPa	SaO2 97,7 %

The v-v ECMO flow was held at maximum values for the entire duration of the procedure, in order to decrease the amount of desaturated blood flow through the lung undergoing lavage. The FiO2 values on the ECMO were determined according to the patient paO2 and varied between 0.6 and 0.8 for the duration of the procedure. It is clear from the initial oxygen values that without the use of the v-v ECMO, the bilateral lung lavage would have caused life threatening hypoxia. The patient’s life would also have been endangered by significant hypercarbia leading to respiratory acidosis. The physiological value of pCO2 was adjusted by changing of gas flow on the ECMO.

After completion of the bilateral lung lavage, the patient was placed on her back and re-intubated with an ordinary endotracheal tube size 7.5. A small amount of remaining fluid was removed from the lungs bronchoscopically. The sedated and hemodynamically stable patient was then moved to the intensive care unit with continued support of the v-v ECMO. In the first few hours post-op, there was a progressive worsening of oxygenation and a left-sided pneumothorax was diagnosed. After the prompt insertion of a chest drain, this was corrected. Due to an insufficient diuresis and risk of hypervolemia, continuous renal replacement therapy (CRRT) was initiated. Within three days, the CRRT and v-v ECMO were discontinued and ventilatory weaning was started On the 9th day, the patient was extubated, but due to poor oxygenation and a hypoxemic index of 200, she was soon placed on non-invasive ventilation. On the 11th day the patient was transferred from the ICU to the Respiratory Care Department for further treatment.

## Conclusion

Pulmonary alveolar proteinosis caused life-threatening respiratory failure in our patient with SaO2 levels below 75 %. The only possibility for treatment was bilateral bronchoalveolar lavage which would rinse the accumulated lipoproteinaceous material out of the lungs using warm saline. Abdelmalak et al. published [2] their experience with high volume wash (up to 50 L in very severe cases) without the need for ECMO and discussed others´approach utilizing hyperbaric oxygen. Noirez et al. presented [3] a 3-step strategy that was used in a patient with PAP-associated refractory hypoxemia and that combined venovenous extracorporeal membrane oxygenation, double-lumen orotracheal intubation, and bilateral multisegmental sequential lavage. In compliance with current literature [2, 3] the oxygenation and ventilation was assured by v-v ECMO and double-lumen orotracheal intubation during the procedure. The oxygenation improved dramatically on v-v ECMO in our patient. In the literature, a few similar cases have been published [5, 6]. The first WLL was performed by Ramirez in 1963 [7]. WLL can be performed with or without ECMO [2, 3, 8]. The WLL procedure could be accompanied by derangements of the acid–base and electrolyte balance and hypervolemia [2, 8]. There is no published information about quantity of absorbed chloride in current literature. In our case, 790 mL of saline was absorbed by the lungs. The plasmatic level of the chloride was checked repeatedly and remained within the physiological range. Since severe generalized oedema of soft tissues was observed together with a change in diuresis, an undefined quantity of chloride is supposed to have been absorbed into the third space. The type of crystalloid to be used in this procedure has not yet been standardized. Derangement of the electrolyte homeostasis causing cardiac arrhythmias could be avoided using Ringer‘s solution [5]. The WLL may also be complicated by pneumotorax [9]. Standardized protocols for WLL have not yet been established. Other methods of treatment are GM-CSF substitutes applied subcutaneously or inhalationally, biological treatment with monoclonal antibodies, plasmapheresis, hyperbaric oxygenoterapy and, in severe cases, even lung transplantation [2, 3, 10]. In our case, we decided for WLL with v-v ECMO support due to the severity of the patient‘s clinical status and with full knowledge of all associated risks. We found this technique to be very effective and, without a doubt, it saved the life of our patient.

Bilateral lung lavage with the support of VV-ECMO is an effective, and often the only feasible, therapeutic method in patients with pulmonary alveolar proteinosis. Quality multidisiplinary teamwork is extremely important.

### Ethics approval

The agreement of the Ethics Committee of University Hospital Motol with this case report´s publication was voiced.

### Statement to confirm

Written informed consent was obtained from the patient for publication of this case report and any accompanying images. A copy of the written consent is available for review by the Editor of this journal.

## Abbreviations

AIDS: acquired immunodeficience virus
BAL: bronchoalveolar lavage
CT: computer tomography
ECMO: extracorporeal membrane oxygenation
GM-CSF: granulocyte-macrophage colony stimulating factor
HRCT: high resolution computer tomography
PAP: pulmonary alveolar proteinosis
PEEP: positive end-expiratory pressure
TIVA: total intravenous anesthesia
VCV: volume-controled ventilation
v-v ECMO: veno-venous extracorporeal membrane oxygenation
WLL: whole-lung lavage
